# Preparation of S_RN_1-Type Coupling Adducts from Aliphatic *gem*-Dinitro Compounds in Ionic Liquids

**DOI:** 10.3390/molecules17054782

**Published:** 2012-04-25

**Authors:** Akio Kamimura, Seiichi Toyoshima

**Affiliations:** Department of Applied Molecular Bioscience, Graduate School of Medicine, Yamaguchi University, Ube 755-8611, Japan

**Keywords:** S_RN_1-type adducts, ionic liquids, nitro compounds, kinetics

## Abstract

S_RN_1-type coupling adducts are readily prepared by the reaction between α-sulfonylesters or α-cyanosulfones and *gem*-dinitro compounds in ionic liquids. The reactions progress smoothly and recovered ionic liquids can be used for several iterations, as long as they are washed with water to remove alkali metallic salts. The reaction rate is slower than the corresponding S_RN_1 reaction in DMSO, but no acceleration on irradiation or no inhibition in the presence of m-DNB are observed.

## 1. Introduction

The S_RN_1 reaction is a unique reaction that proceeds via a single electron transfer process [1,2,3]. The reaction usually starts with a single electron transfer that generates a radical anion species, which then gives a radical species via cleavage of the anion radical. Then, the radical reacts with a coupling partner to form products. The reaction is usually performed in either liquid ammonia or a dipolar aprotic solvent such as DMSO and HMPA. The reaction progresses through a radical chain mechanism and the reaction rate are significantly lowered by the presence of small amounts of a radical inhibitor such as *p*-dinitrobenzene. The S_RN_1 reaction is frequently used to construct aromatic compounds [4,5,6,7,8,9,10,11,12,13,14,15]. The S_RN_1 reaction between aliphatic compounds produces a new carbon-carbon or carbon-heteroatom bond between sterically hindered carbons in good yields. This type of bond formation is usually not easily achieved using any other reactions in organic synthesis. The adducts from an aliphatic S_RN_1 reaction are regarded as precursors for further palladium coupling materials [16] or tri- or tetrasubstituted alkenes [17,18,19,20,21,22].

Recently ionic liquids have attracted significant interest in organic synthesis because of their unique properties such as wide redox windows, high polarities and high solubilities [23,24,25,26,27,28]. During the course of our investigation on ionic liquid chemistry [29,30,31], it occurred to us that ionic liquids could become a new solvent system for a reaction via electron transfer such as S_RN_1 reaction. To our best of knowledge, there have been no reports that employ ionic liquids for such reactions. In this paper, we demonstrate that the S_RN_1-type coupling adducts are indeed readily obtained in the reaction in ionic liquids. 

## 2. Results and Discussion

**Scheme 1:**
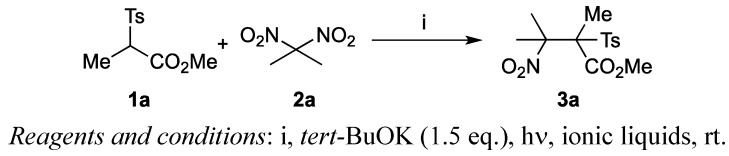
S_RN_1-type coupling reactions between **1a** and **2a**.

We first examined various ionic liquids for the S_RN_1-type coupling reaction between *gem*-dinitro compounds and α-sulfonylesters. *gem*-Dinitropropane **2a** was added to a mixture of *tert*-BuOK and α-sulfonyl propionic ester **1a** in different ionic liquids under photoirradiation produced by a usual tungsten lamp, and the desired coupling product **3a** was isolated (Scheme 1) [32]. Table 1 summarizes the results.

**Table 1:** S_RN_1-type coupling reactions of **1a** in various ionic liquids.

Entry	Ionic liquids ^a^	Time (h)	3a; yield (%) ^b^
1	[bmim][PF_6_]	11	58 (13)
2	[bmim][BF_4_]	7	71 (23)
3	[bmim][NTf_2_]	7	55 (5)
4	[PP13][NTf_2_]	4	66
5	[TMPA][NTf_2_]	7	76
6	[DEME][BF_4_]	6	58 (17)

^a^ [bmim]:1-butyl-3-methyimidazolium; [PP13]:1-methyl-1-propylpiperidinium; [TMPA]: propyltrimethylammonium; [DEME]; *N,N*-diethyl-*N*-methyl-*N*-(2-methoxyethyl)ammonium; ^b^ Isolated yields. Recovery of **1a** is in parentheses.

**Scheme 2:**
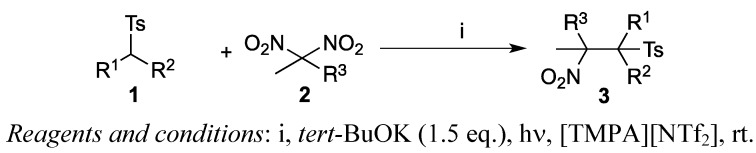
S_RN_1-type coupling reaction in ionic liquids.

The coupling reaction between **1a** and **2a** took place smoothly in ionic liquids to give **3a** in good yield. For example, the reaction in [bmim][PF_6_] resulted in the formation of the coupling product **3a** in 58% yield (entry 1). The reaction was complete after 11 h at room temperature, but some amounts of the starting material **1a** remained and were recovered from the reaction mixture. Although we have examined many bases such as Me_4_NOH, DBU and Et_3_N, none of these amine bases worked well in the reaction. Starting material **1a** was recovered completely. Use of other ionic liquids that contained BF_4_ and NTf_2_ as a counter anion were examined (entries 2–6). The reaction progressed smoothly and the corresponding adduct **3a** was isolated in good yield. Thus, ionic liquids were useful solvents for the coupling reaction. The reaction for other starting materials was explored next (Scheme 2). Table 2 summarizes the results. A mixture of **1b** and 2,2-dinitropropane (**2a**) in [TMPA][NTf_2_], for example, afforded coupling adduct **3b** in 69% yield (entry 1). The reaction was complete within 8 to 24 h. Coupling with 2,2-dinitrobutane (**2b**) also gave the products in a 1:1 mixture of two possible diastereomers (entries 2 and 3). α-Cyanosulfonyl compounds also underwent the reaction in ionic liquid, producing the corresponding coupling products **3e** to **3h** in moderate to good yields (entries 4–7). Thus, ionic liquids are useful solvents for promoting the coupling reaction effectively.

**Table 2:** The coupling reactions with various sulfonyl compounds **1**.

Entry	1	R^1^	R^2^	R^3^	Time (h)	3; Yield (%) ^a^
1	**1b**	Me	CO_2_Et	Me	8	**3b**; 69
2	**1a**	Me	CO_2_Me	Et	22	**3c**; 68
3	**1b**	Me	CO_2_Et	Et	20	**3d**; 66
4	**1c**	Me	CN	Me	24	**3e**; 50
5	**1d**	Et	CN	Me	24	**3f**; 75
6	**1e**	C_4_H_9_	CN	Me	21	**3g**; 47
7	**1f**	CH_2_=CH(CH_2_)_3_–	CN	Me	21	**3h**; 44

^a^ Isolated yields.

**Scheme 3:**
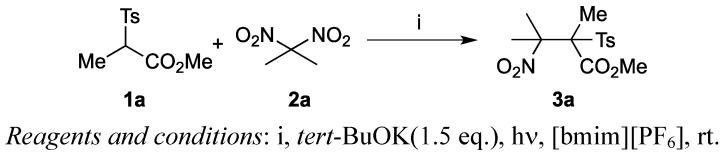
Iteration use of ionic liquids for S_RN_1-type coupling reaction.

The repeated use of ionic liquids was examined for the reaction between **1a** and **2a** in [bmim][PF_6_] (Scheme 3). Table 3 summarizes the results.

**Table 3:** Recycling use of [bmim][PF_6_] for the coupling reaction to give **3a**.

Times	Time(h)	3a; Yield (%)^ a^
1	11	58
2	7	62
3	7	19
4	7	51 ^b^
5	7	50 ^b^

^a^ Isolated yields; ^b^ The washing treatment of ionic liquid was carried out before the reaction.

The recycling of the ionic liquids was performed in the following way: after the first reaction was completed, we performed a usual work-up. Thus, product **3a** was isolated in 58% yield by direct extraction with ether from the ionic liquid and the remaining [bmim][PF_6_] was used directly for the next reaction. The second run worked well and **3a** was prepared in 62% yield. The third run, however, occurred sluggishly, and the desired product **3a** was isolated in only 19% yield. We thought this might be due to accumulating side products such as sodium nitrite. Therefore, [bmim][PF_6_] was washed with water to remove salts and other water-soluble impurities that had accumulated during the reaction. The ionic liquid was recovered without significant loss. After drying, we used the recovered [bmim][PF_6_] for the reaction and obtained **3a** in 51% yield. When we used it for the fifth time, [bmim][PF_6_] worked well and product **3a** was isolated in 50% yield. Thus, the present procedure allowed us to use the ionic liquid several times. We examined iterative use of ionic liquids [TMPA][NTf_2_] for the reaction and successfully obtained **3a** in good to moderate yields (Table 4). High yields of **3a** were achieved until six times use, when **3a** was obtained in 60%, after then the yields decreased to less than 40%. 

**Table 4:** Coupling reactions of compounds **1a** with iterative use of [TMPA][NTf_2_].

Times	1	2	3	4	5	6	7	8	9	10
**3a**; Yield (%) ^a^	86	87	80	78	68	60	40	32	30	24

^a^ Isolated yields. The washing treatment of ionic liquid was carried out for each time.

**Figure 1:**
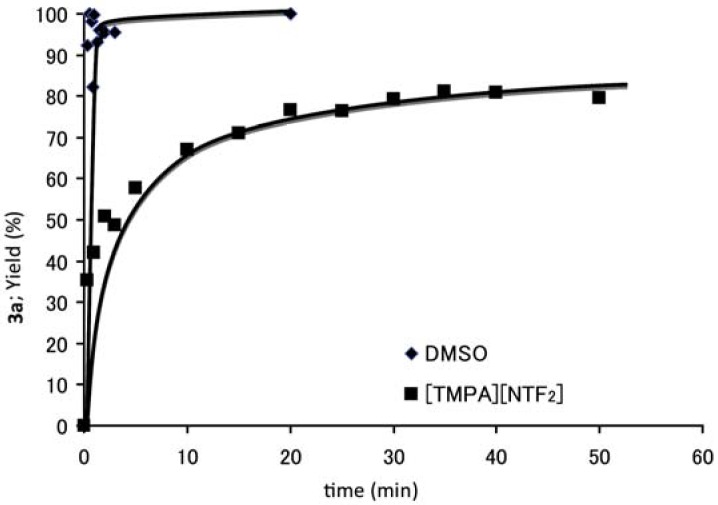
Time course of the S_RN_1 reaction of **1a** and **2a**.

To explore the reaction profile, we examined the reaction kinetics. Figure 1 shows the comparison of the reaction between **1a** and **2a** under classical conditions employing DMSO as a solvent and under the present conditions using [TMPA][NTf_2_] as a solvent.

Kinetic measurements were performed for the reaction on a 0.2 M scale. Thus, the mixture of 0.6 mmol of α-tosylpropionate **1a** and 2,2-dinitropropane (**2a**) in DMSO or [TMPA][NTf_2_] (2.5 mL) was used for the kinetic measurements. We detected product **3a** by HPLC analyses and estimated it using the curve fitting method. The reaction in DMSO progressed very fast to give **3a** almost quantitatively within a minute, while the reaction in [TMPA][NTf_2_] progressed much slowly, and the yield of **3a** increased to greater than 80% after about 40 minutes. This difference in the reaction rate should arise from the difference in viscosity because ionic liquids usually possess greater viscosities than any other usual organic solvent [33]. 

**Scheme 4:**
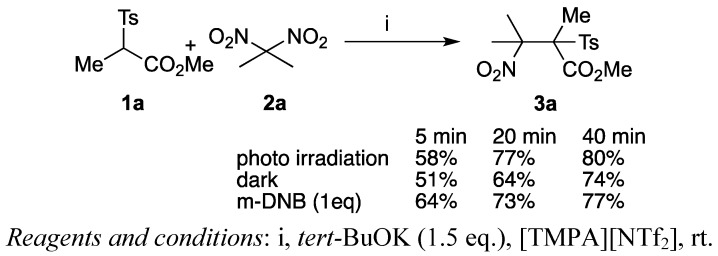
Photo irradiation and additive effects.

It is noteworthy that the coupling reaction in ionic liquids showed no inhibition by additives or acceleration by photoirradiation. Scheme 4 summarizes the results in which the reaction progress was almost the same even on addition of *m*-dinitrobenzene or with irradiation from a tungsten lamp. These results are in sharp contrast with the conventional S_RN_1 reaction in which inhibition by adding aromatic nitro compounds and acceleration with photo irradiation have been clearly observed [32].

## 3. Experimental

### General

All ^1^H- and ^13^C-NMR spectra were measured in CDCl_3_ and recorded on a JEOL Lamda-500 spectrometer (at 500 MHz for ^1^H and 126 MHz for ^13^C). All reactions were performed under a nitrogen atmosphere unless otherwise mentioned. DMSO was dried over CaH_2_ and distilled under reduced pressure before use. Ionic liquids, except for [DEME][NTf_2_], were purchased from Kanto Chemical Co. Ltd. [DEME][NTf_2_] was supplied by Nisshinbo Co. Ltd. Photoirradiation was carried out by a standard 40 W tungsten lamp. Elemental analyses and high-resolution mass spectra were measured at Tokiwa Instrumental Analysis Center, Yamaguchi University, Ube, Japan.

*Methyl 2*,*3-dimethyl-3-nitro-2-tosylbutanoate* (**3a**): Under a nitrogen atmosphere, *t*-BuOK (102.2 mg, 0.91 mmol) was added to a solution of **1a** (147.9 mg, 0.61 mmol) in [TMPA][NTf_2_] (2.5 mL) at room temperature. Then 2,2-dinitropropane (91.0 mg, 0.68 mmol) was added and the reaction mixture was stirred at room temperature for 7 h under irradiation by a fluorescent light (365 nm). The reaction mixture was extracted with ether (3 mL × 30) and the combined organic phase was washed with 1 M HCl (10 mL) and saturated NaCl (20 mL). The organic phase was dried over Na_2_SO_4_, filtered, and concentrated *in vacuo*. The residue was purified by flash chromatography (silica gel, 10:1, 8:1, and then 5:1 hexane-EtOAc) to give **3a** (153.3 mg, 465.4 mmol, 76%) as a white solid; mp. 101.5–102.5 °C; ^1^H-NMR (CDCl_3_) δ 7.70 (dd, 2 H, *J* = 13.4, 8.5 Hz), 7.29 (dd, 2 H, *J* = 10.9, 4.7 Hz), 3.80–3.42 (m, 3 H), 2.44 (s, 3 H), 2.26 (s, 3 H), 1.97 (s, 3 H), 1.66 (s, 3 H); ^13^C-NMR (CDCl_3_) δ 166.85, 146.21, 133.53, 130.90, 129.59, 93.80, 76.20, 53.40, 26.44, 25.23, 21.83, 18.25; Anal. Calcd. for C_14_H_19_NO_6_S: C, 51.05; H, 5.81; N, 4.25%. Found: C, 50.91; H, 5.85; N, 4.32%.

*Ethyl2*,*3-dimethyl-3-nitro-2-tosylbutanoate* (**3b**): Isolated as an oil (175.2 mg, 69%); ^1^H-NMR (CDCl_3_). 7.68 (d, 2 H, *J* = 8.4 Hz), 7.31 (d, 2 H, *J* = 8.6 Hz), 4.13–3.94 (m, 2 H), 2.42 (s, 3 H), 2.26 (s, 3 H), 1.95 (s, 3 H), 1.66 (s, 3 H), 1.12 (t, 3 H, *J* = 7.2 Hz); ^13^C-NMR (CDCl_3_). 166.23, 146.13, 133.56, 130.97, 129.50, 93.69, 76.11, 62.87, 26.36, 25.44, 21.79, 18.35, 13.57; HRMS (ESI^+^ M+NH_4_)^+^ *m/z* 361.1438. Calcd. for C_15_H_25_N_2_O_6_S 361.1433.

*Methyl2*,*3-dimethyl-3-nitro-2-tosylpentanoate* (**3c**): Isolated as a white solid (116.9 mg, 68%, 1:1 inseparable diastereomeric mixture); mp. 136–137 °C; ^1^H-NMR (CDCl_3_). 7.72 (dd, 2 H for one isomer, *J* = 8.4, 1.8 Hz), 7.67 (dd, 2 H for another isomer, *J* = 8.3, 1.6 Hz), 7.35–7.30 (m, 2 H for both isomers), 3.66 (s, 3 H for one isomer), 3.51 (s, 3 H for 1 isomer), 2.85 (dq, 1 H for one isomer, *J* = 14.6, 7.3 Hz), 2.68 (dq, 1 H for another isomer, *J* = 14.9, 7.4 Hz), 2.44–2.40 (m, 1 H for another isomer), 2.44 (s, 3 H for one isomer), 2.43 (s, 3 H for another isomer), 2.35 (dq, 1 H for one isomer, *J* = 14.2, 7.0 Hz), 2.20 (s, 3 H one isomer), 1.94 (s, 3 H for another isomer), 1.78 (s, 3 H for one isomer), 1.61 (s, 3 H for another isomer), 0.95 (t, 3 H for one isomer, *J* = 7.2 Hz), 0.83 (t, 3 H for another isomer, *J* = 6.4 Hz); ^13^C-NMR (CDCl_3_). 166.92, 166.35, 146.19, 146.06, 133.93, 133.90, 130.99, 129.64, 129.47, 98.47, 96.10, 77.16, 76.90, 53.48, 53.38, 29.91, 29.89, 21.82, 20.29, 18.86, 18.53, 17.53, 9.21, 8.78; HRMS (ESI^+^ M+NH_4_) *m/z* = 361.1437. Calcd. for C_15_H_25_N_2_O_6_S 361.1433.

*Ethyl2*,*3-dimethyl-3-nitro-2-tosylpentanoate* (**3d**): Isolated as an oil (135.4 mg, 66%, 1:1 inseparable diastereomeric mixture); ^1^H-NMR (CDCl_3_). 7.74 (d, 2 H for one isomer, *J* = 8.4 Hz), 7.69 (d, 2 H for another isomer, *J* = 8.4 Hz), 7.34 (dd, 2 H for one isomer, *J* = 3.7, 0.6 Hz), 7.32 (d, 2 H for another isomer, *J* = 3.7 Hz), 4.19–4.08 (m, 2 H for one isomer), 4.00–3.89 (m, 2 H for another isomer), 2.95–2.83 (m, 1 H for one isomer), 2.69 (dq, 1 H for another isomer, *J* = 14.9, 7.4 Hz), 2.45 (s, 3 H for one isomer), 2.44 (s, 3 H for another isomer), 2.44–2.37 (m, 1 H for one isomer), 2.37–2.28 (m, 1 H for another isomer), 2.23 (s, 3 H for one isomer), 1.95 (s, 3 H for another isomer), 1.78 (s, 3 H for one isomer), 1.62 (s, 3 H for another isomer), 1.20 (t, 3 H for one isomer, *J* = 7.2 Hz), 1.06 (t, 1 H for one isomer, *J* = 7.2 Hz), 0.95 (t, 3 H for another isomer, *J* = 7.3 Hz), 0.84 (t, 3 H for another isomer, *J* = 7.4 Hz); ^13^C-NMR (CDCl_3_). 166.38, 165.72, 146.07, 145.97, 134.04, 133.87, 131.13, 131.09, 129.53, 129.40, 98.46, 96.01, 77.63, 77.08, 63.16, 62.74, 30.19, 29.85, 21.82, 21.80, 20.33, 19.02, 18.57, 17.50, 13.60, 13.55, 9.21, 8.79; HRMS (ESI^+^ M+H) *m/z* = 375.1569. Calcd. for C_16_H_27_N_2_O_6_S 375.1590.

*2*,*3-Dimethyl-3-nitro-2-tosylbutanenitrile* (**3e**): Isolated as a white solid (174.1 mg, 50%) mp. 75–76 °C; ^1^H-NMR (CDCl_3_). 7.92 (d, 2 H, *J* = 8.4 Hz), 7.44 (d, 2 H, *J* = 8.0 Hz), 2.50 (s, 3 H), 2.15 (s, 3 H), 1.98 (s, 3 H), 1.69 (s, 3 H); ^13^C-NMR (CDCl_3_). 147.66, 131.54, 130.11, 130.09, 116.13, 90.97, 66.61, 25.82, 22.43, 22.00, 19.27; Anal. Calcd. for C_13_H_16_N_2_O_4_S: C, 52.69; H, 5.44; N, 9.45%. Found: C, 52.74; H, 5.38; N, 9.11%.

*2-(2-Nitropropan-2-yl)-2-tosylhexanenitrile* (**3f**): Isolated as a white solid (142.5 mg, 75%); mp. 68–70 °C; ^1^H-NMR (CDCl_3_). 7.89 (d, 2 H, *J* = 8.3 Hz), 7.43 (d, 2 H, *J* = 8.1 Hz), 2.49 (s, 3 H), 2.36 (dq, 1 H, *J* = 15.0, 7.5 Hz), 2.09 (s, 3 H), 1.95 (s, 3 H), 1.91 (dq, 1 H, *J* = 15.1, 7.5 Hz), 0.87 (t, 3 H, *J* = 7.5 Hz); ^13^C-NMR (CDCl_3_). 147.51, 132.70, 131.43, 130.11, 114.90, 91.81, 72.66, 25.81, 25.22, 23.29, 22.00, 11.50; HRMS (ESI^+^ M+NH_4_) *m/z* = 328.1360. Calcd. for C_14_H_21_N_3_O_4_S 328.1331.

*2-Ethyl-3-methyl-3-nitro-2-tosylbutanenitrile* (**3g**): Isolated as a white solid (110.8 mg, 47%); mp. 72.8–73.0 °C; ^1^H-NMR (CDCl_3_). 7.91 (d, 2 H, *J* = 8.4 Hz), 7.43 (d, 2 H, *J* = 8.1 Hz), 2.49 (s, 3 H), 2.23 (ddd, 1 H, *J* = 15.2, 12.4, 4.3 Hz), 2.10 (s, 3 H, s), 1.96 (3 H, s), 1.79 (1 H, ddd, *J* 15.2, 12.6, 5.0), 1.38–1.08 (3 H, m), 0.93–0.82 (1 H, m) and 0.77 (3 H, t, *J* 7.3); ^13^C-NMR (CDCl_3_). 147.53, 132.63, 131.44, 130.05, 115.04, 92.03, 71.90, 31.11, 29.01, 25.97, 23.04, 22.66, 21.99 and 13.53. Anal. Calcd. for C_16_H_22_N_2_O_4_S: C, 56.78; H, 6.55; N, 8.28%. Found: C, 56.86; H, 6.44; N, 8.27%.

*2-(2-Nitropropan-2-yl)-2-tosylhept-6-enenitrile* (**3h**): Isolated as a white solid (115.9 mg, 44%); mp. 74–75 °C; ^1^H-NMR (CDCl_3_). 7.90 (d, 2 H, *J* = 8.4 Hz), 7.42 (d, 2 H, *J* = 8.1 Hz), 5.55 (ddt, 1 H, *J* = 17.0, 10.3, 6.7 Hz), 4.96 (dt, 1 H, *J* = 10.9, 1.7 Hz), 4.93 (dq, 1 H, *J* = 17.2, 1.6 Hz), 2.49 (s, 3 H), 2.23 (ddd, 1 H, *J* = 15.3, 12.6, 4.4 Hz), 2.09 (s, 3 H), 1.95 (s, 3 H), 1.99–1.95 (m, 1 H), 1.93–1.85 (m, 1 H), 1.79 (ddd, 1 H, *J* = 15.3, 12.6, 5.0 Hz), 1.50–1.38 (m, 1 H), 1.10–0.94 (m, 1 H); ^13^C-NMR (CDCl_3_). 147.59, 136.60, 132.53, 131.48, 130.08, 116.30, 115.00, 92.02, 71.81, 33.23, 30.66, 26.00, 25.93, 23.07, 21.99. Anal. Calcd. for C_17_H_22_N_2_O_4_S: C, 58.27; H, 6.33; N, 7.99%. Found: C, 58.20; H, 6.30; N, 7.94%.

## 4. Conclusions

We have demonstrated the first examples of S_RN_1-type coupling reactions in an ionic liquid, which not only possesses high polarity but is also regarded as a good solvent for promoting the electron transfer process. The ionic liquids [bmim][PF_6_] and [TMPA][NTf_2_] were useful for the efficient progress of the reaction. Although amine base was not effective for the progress of the reaction, *t*-BuOK was a useful base to enhance the reactions. Although the reaction rate in the S_RN_1 reaction was not as fast as that in the conventional S_RN_1 reaction in DMSO, ionic liquids have an advantage over the conventional method because of the reusability of the solvent if it was washed with water; a simple manipulation that enabled the ionic liquid to be reused for another reaction. A notable contrast from the conventional S_RN_1 reaction in DMSO was the fact that the reaction was not impeded by the presence of *m*-DNB, and the coupling products were obtained in a similar yield. The use of ionic liquids for other reactions is now under investigation in our laboratory.

## Supplementary Materials

Supplementary materials can be accessed at: http://www.mdpi.com/1420-3049/17/5/4782/s1.
