# Supplementary material for: Preparation of SRN1-Type Coupling Adducts from Aliphatic gem-Dinitro Compounds in Ionic Liquids
Source: Molecules. 2012 Apr 25;17(5):4782–90. doi: 10.3390/molecules17054782 (PMC6268892; doi:10.3390/molecules17054782)
Supplement: Supplementary file 1 [file molecules-17-04782-s001.pdf]

Supporting Information for

Preparation of $S_{RN}1$-type coupling adducts from aliphatic *gem*-dinitro compounds in ionic liquids

Akio Kamimura* and Seiichi Toyoshima

Department of Applied Molecular Bioscience, Graduate School of Medicine, Yamaguchi University, Ube 755-8611 Japan

E-mail: ak10@yamaguchi-u.ac.jp

Table of contents

^{1}H and ^{13}C NMR for compounds **3a** – **3h** page 2

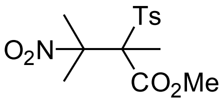
Ts
O2N
CO2Me

3a

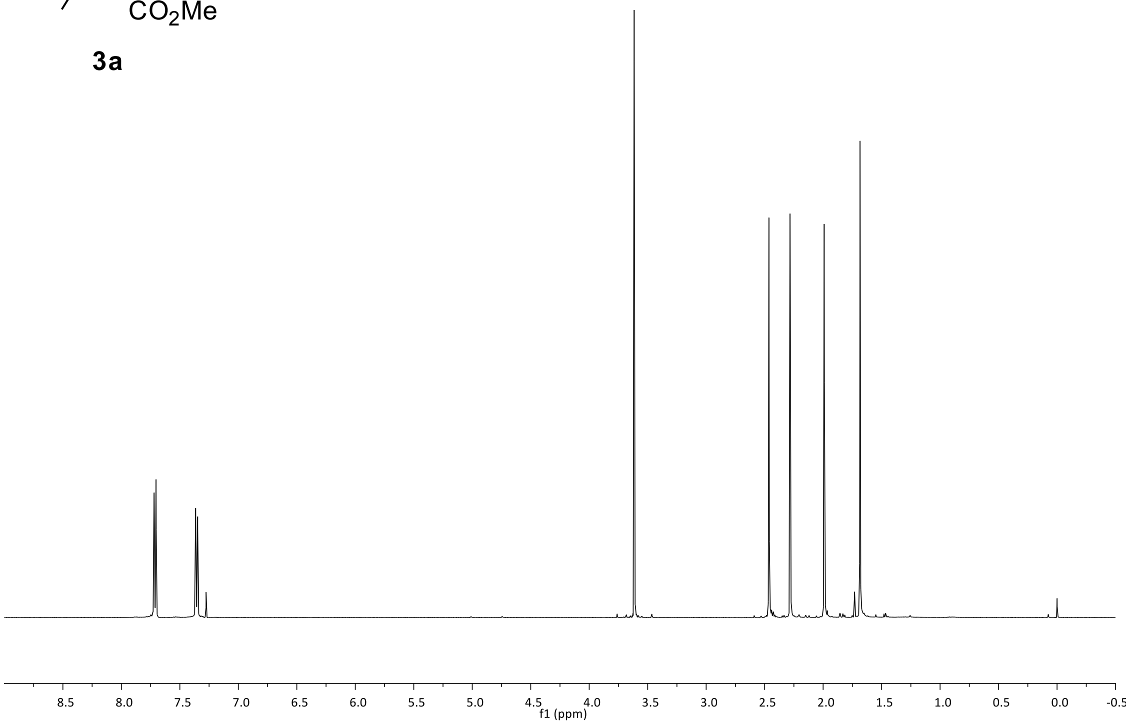
8.5 8.0 7.5 7.0 6.5 6.0 5.5 5.0 4.5 4.0 3.5 3.0 2.5 2.0 1.5 1.0 0.5 0.0 -0.5
f1 (ppm)

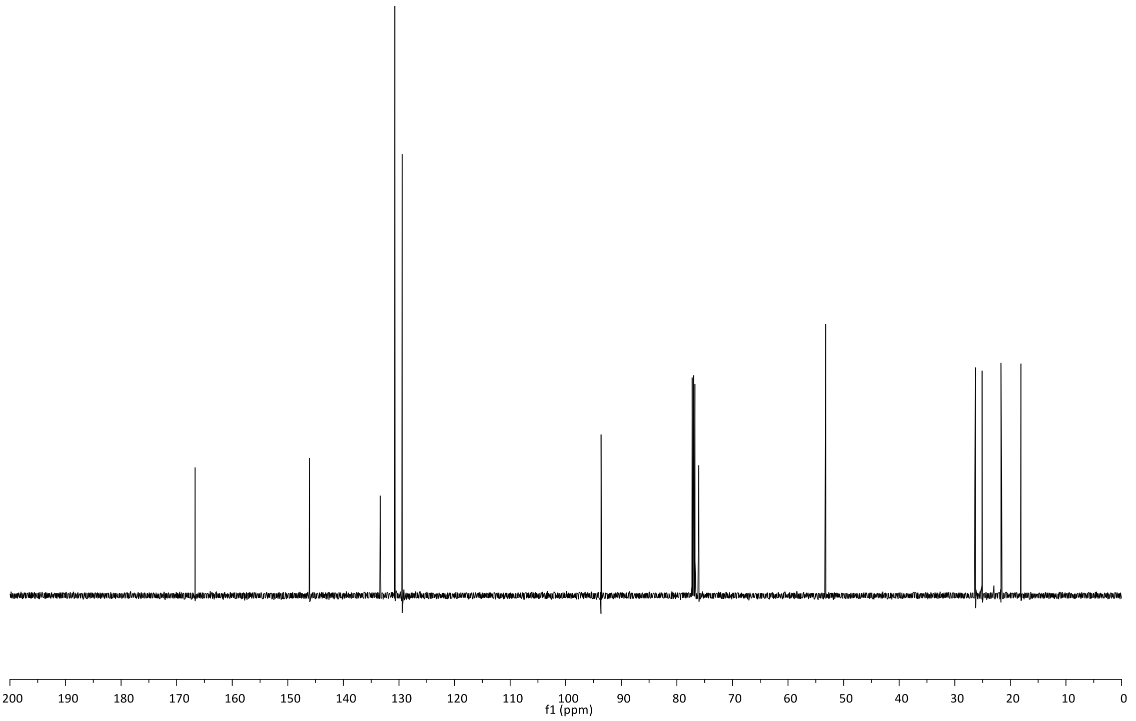
200 190 180 170 160 150 140 130 120 110 100 90 80 70 60 50 40 30 20 10 0
f1 (ppm)

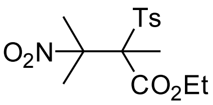
Ts
O2N
CO2Et

3b

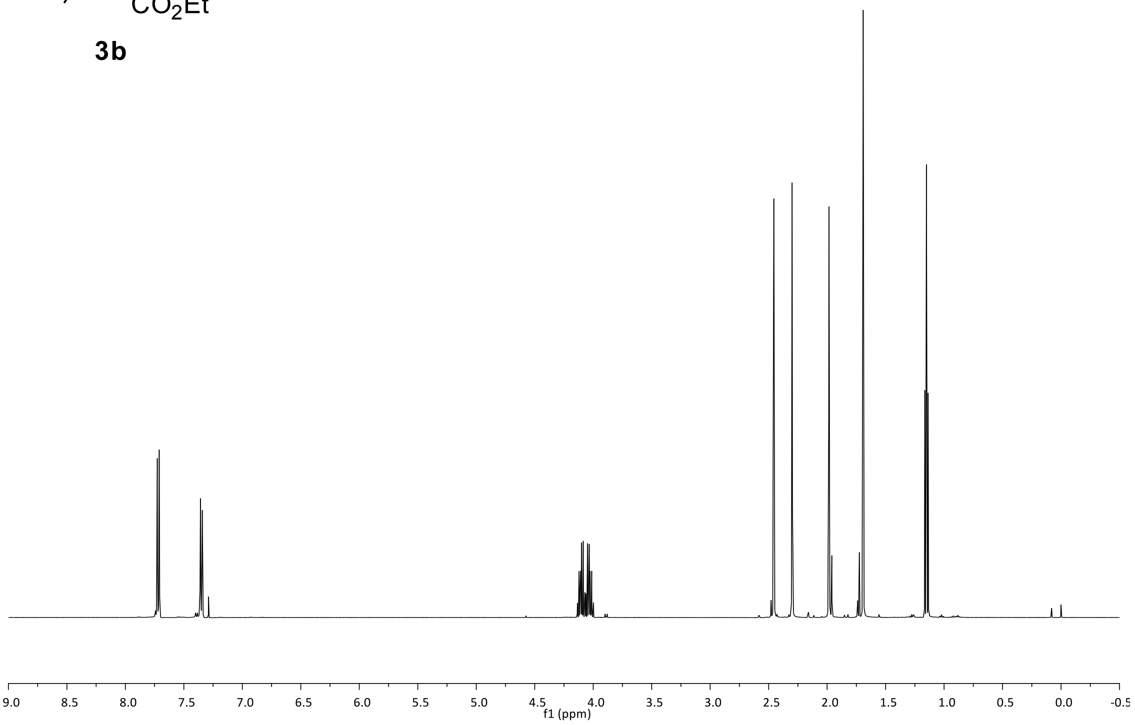
9.0
8.5
8.0
7.5
7.0
6.5
6.0
5.5
5.0
4.5
4.0
3.5
3.0
2.5
2.0
1.5
1.0
0.5
0.0
f1 (ppm)

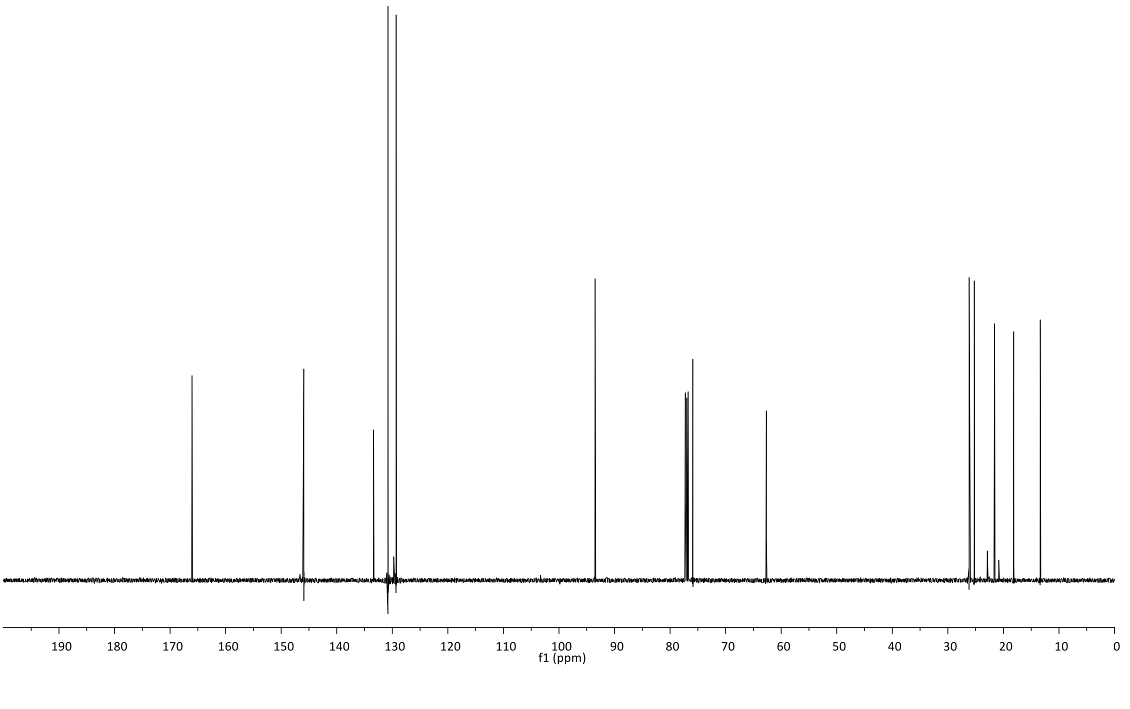
190
180
170
160
150
140
130
120
110
100
90
80
70
60
50
40
30
20
10
0
f1 (ppm)

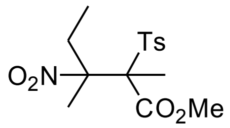

3c

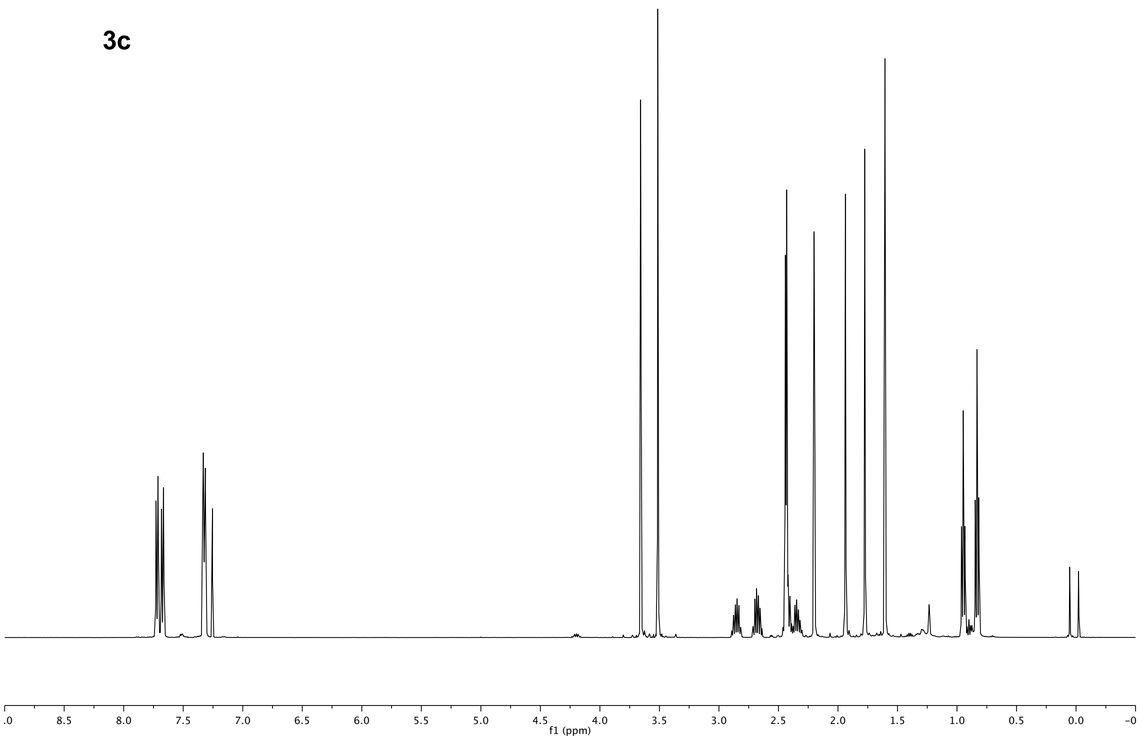

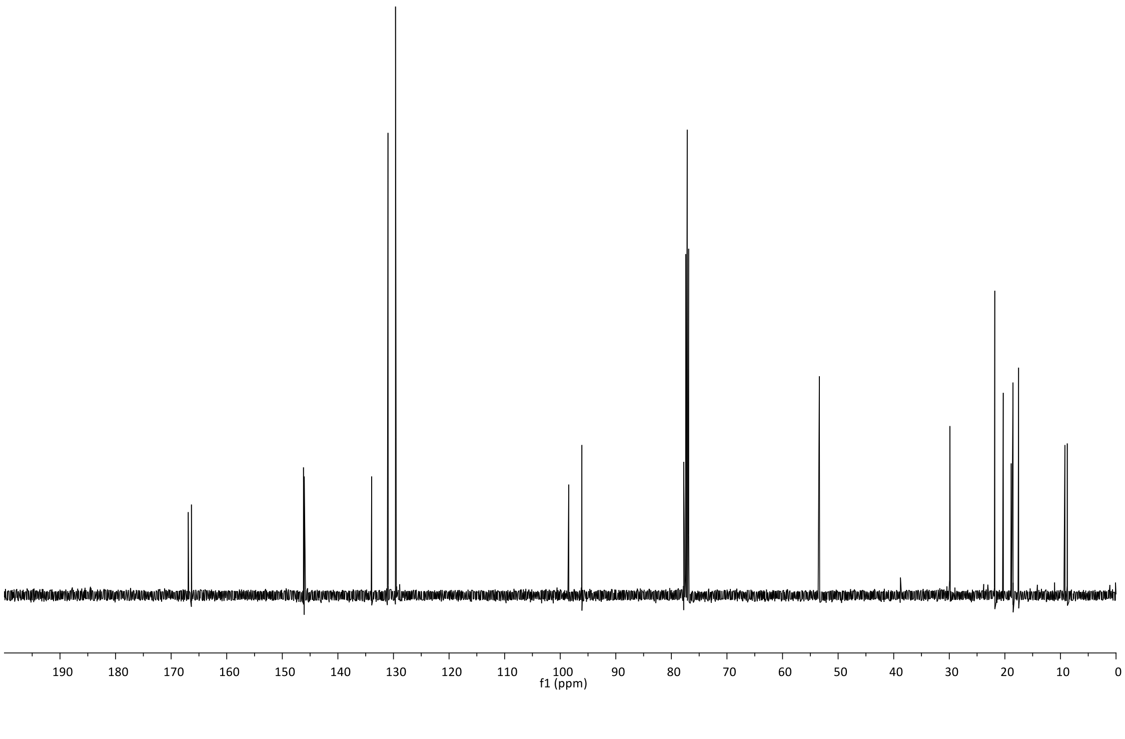

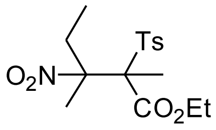
Ts
O2N
CO2Et

3d

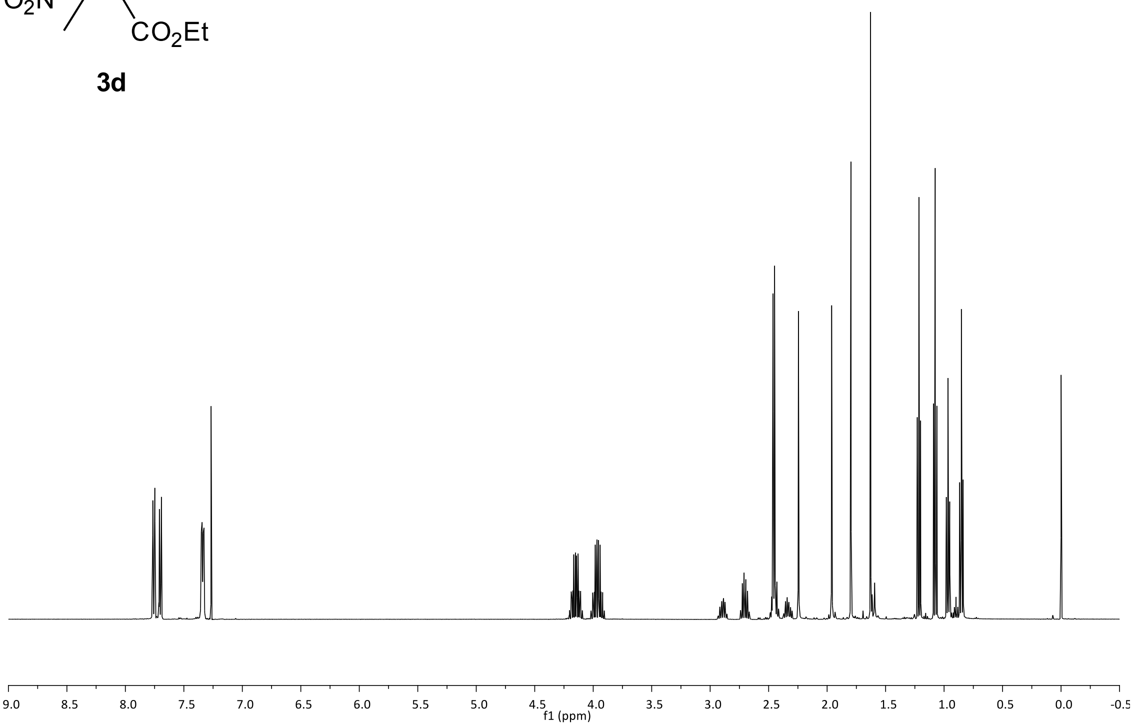
9.0 8.5 8.0 7.5 7.0 6.5 6.0 5.5 5.0 4.5 4.0 3.5 3.0 2.5 2.0 1.5 1.0 0.5 0.0 -0.5
f1 (ppm)

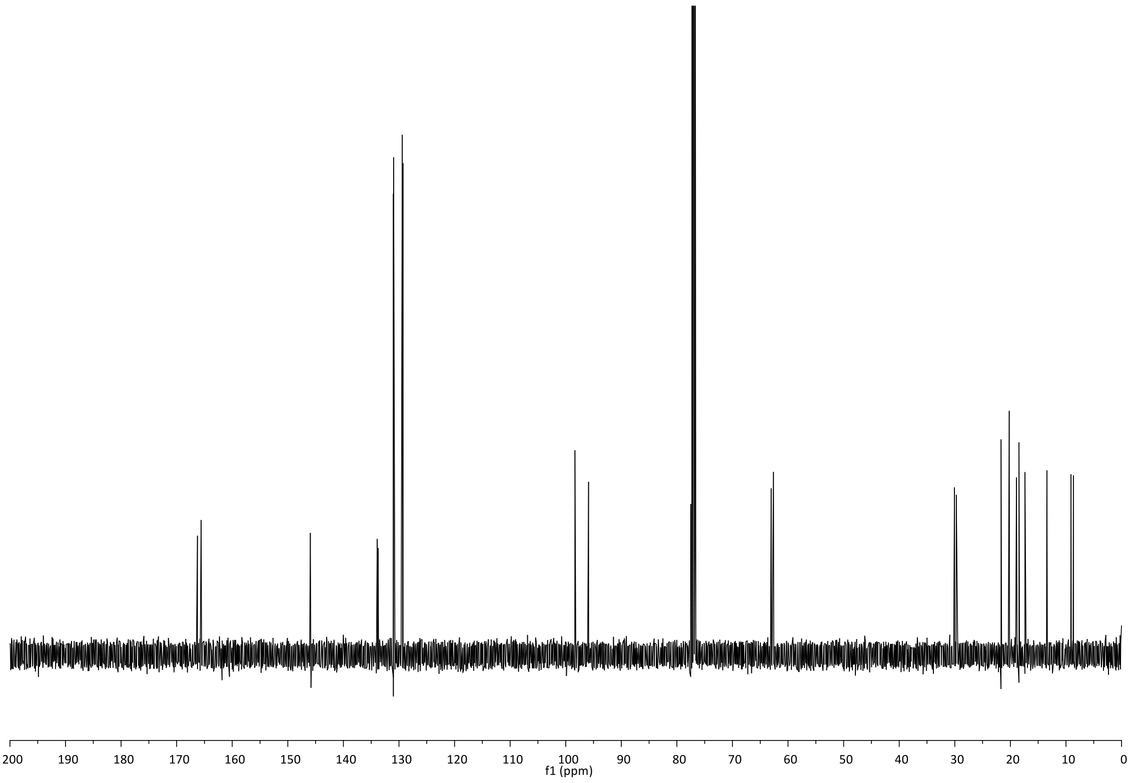
200 190 180 170 160 150 140 130 120 110 100 90 80 70 60 50 40 30 20 10 0
f1 (ppm)

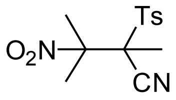
Ts
O2N
CN

3e

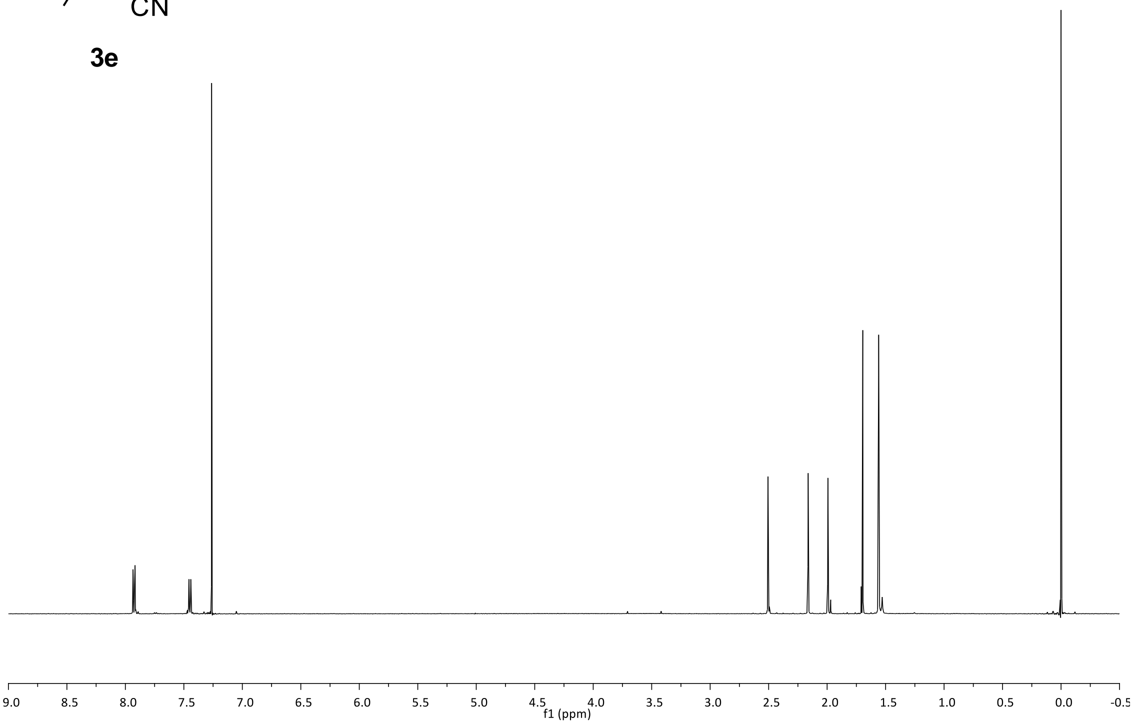
9.0 8.5 8.0 7.5 7.0 6.5 6.0 5.5 5.0 4.5 4.0 3.5 3.0 2.5 2.0 1.5 1.0 0.5 0.0 -0.5
f1 (ppm)

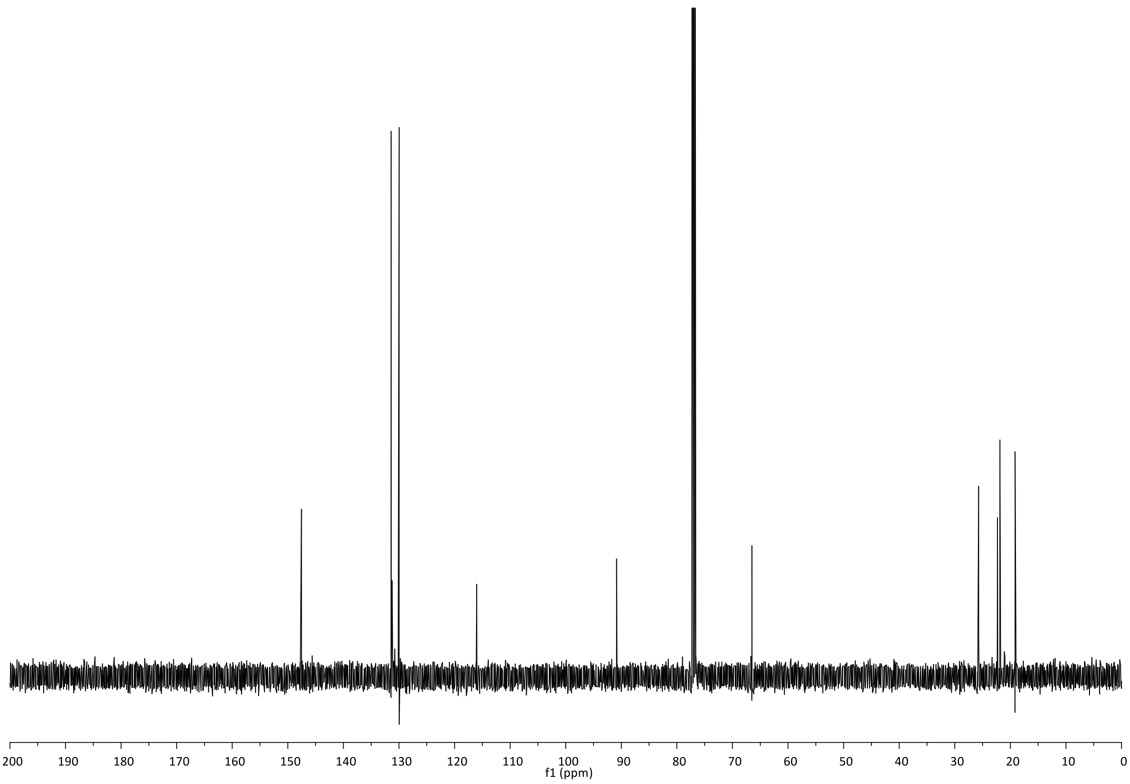
200 190 180 170 160 150 140 130 120 110 100 90 80 70 60 50 40 30 20 10 0
f1 (ppm)

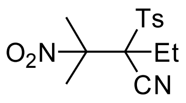

Ts
O_2N
Et
CN

3f

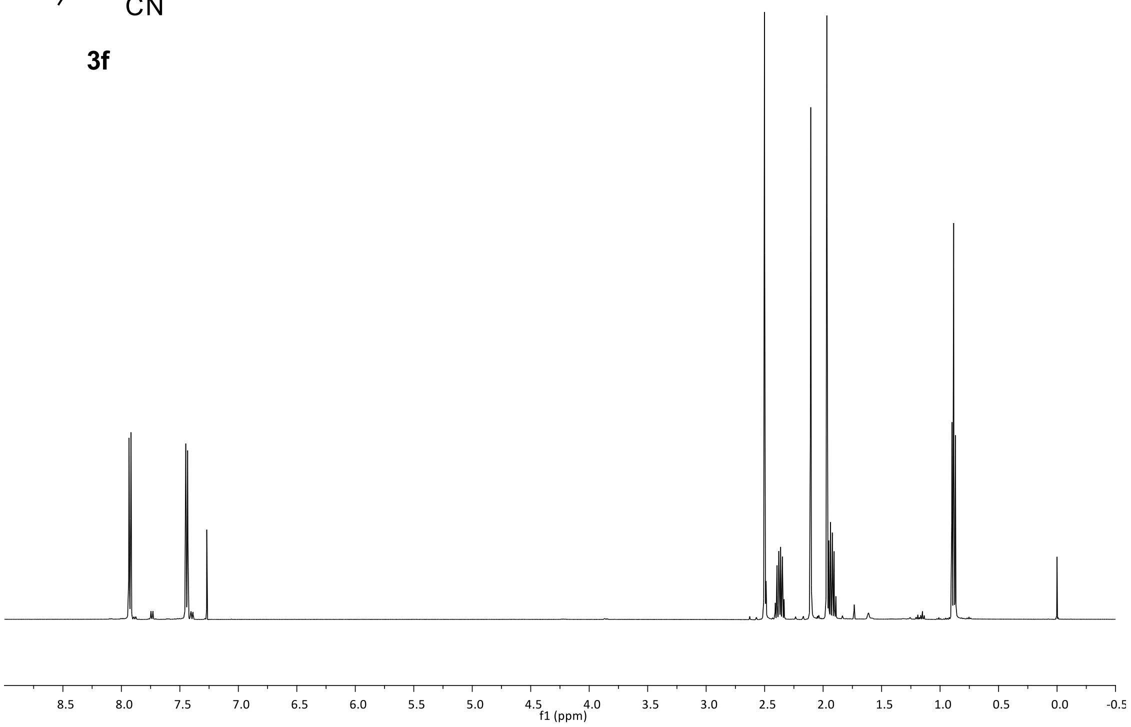

8.5
8.0
7.5
7.0
6.5
6.0
5.5
5.0
4.5
4.0
3.5
3.0
2.5
2.0
1.5
1.0
0.5
0.0
f1 (ppm)

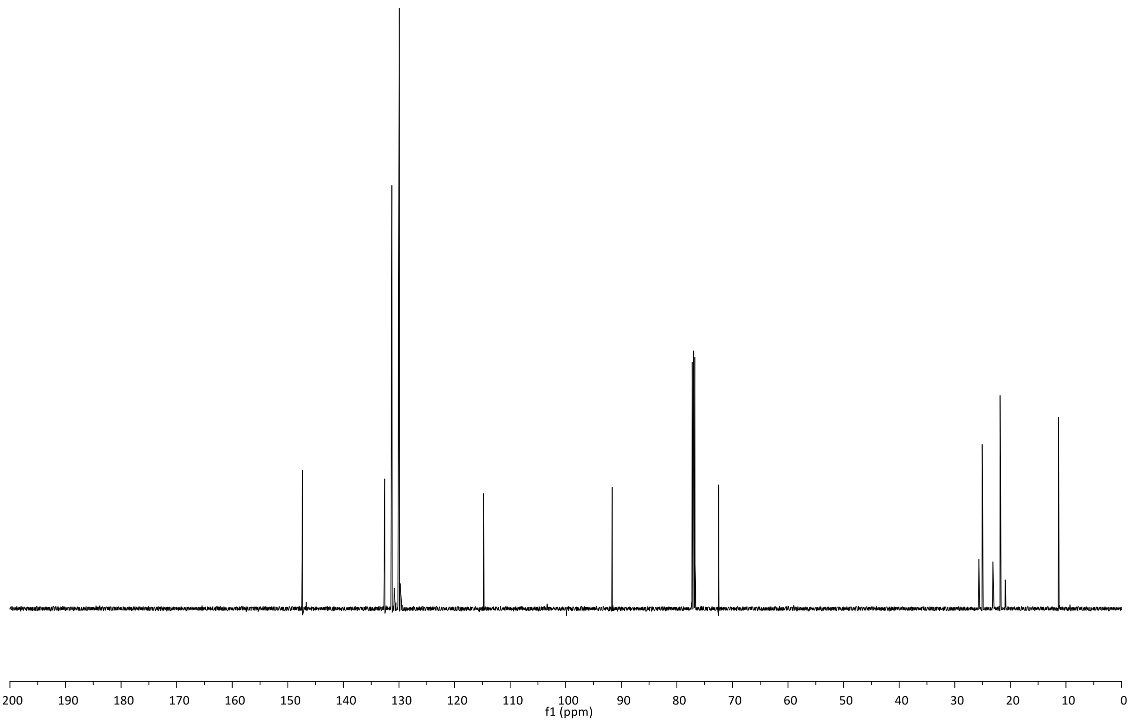

200
190
180
170
160
150
140
130
120
110
100
90
80
70
60
50
40
30
20
10
0
f1 (ppm)

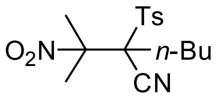
Ts
O2N
n-Bu
CN

3g

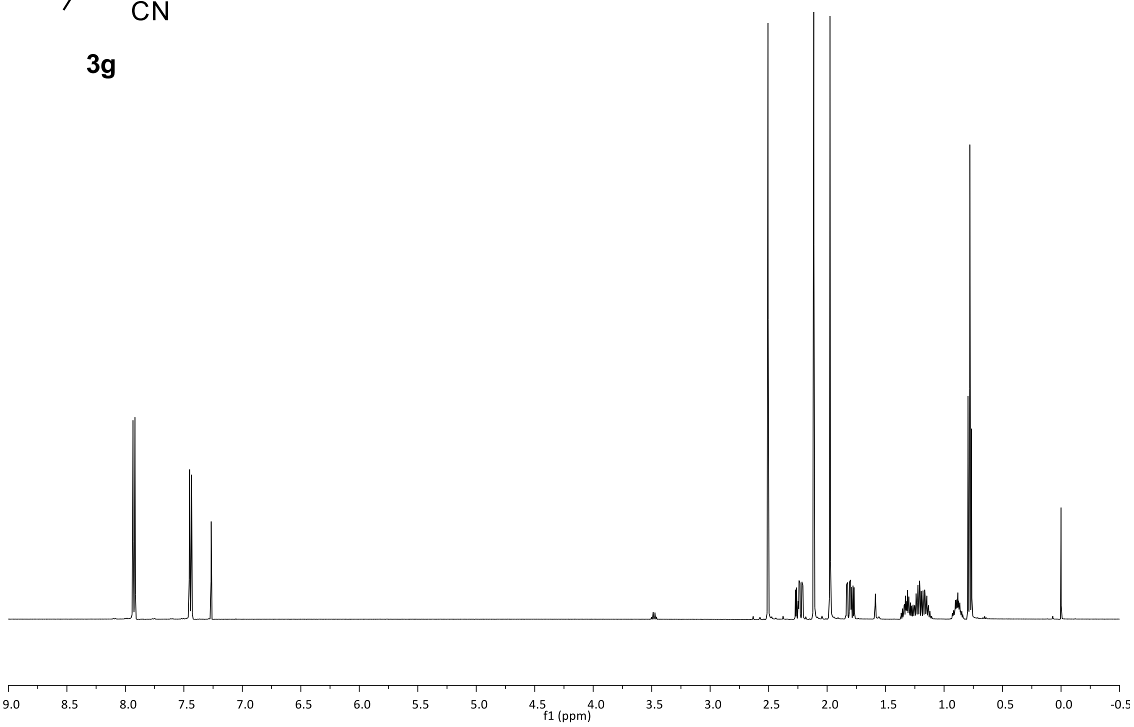
9.0
8.5
8.0
7.5
7.0
6.5
6.0
5.5
5.0
4.5
4.0
3.5
3.0
2.5
2.0
1.5
1.0
0.5
0.0
f1 (ppm)

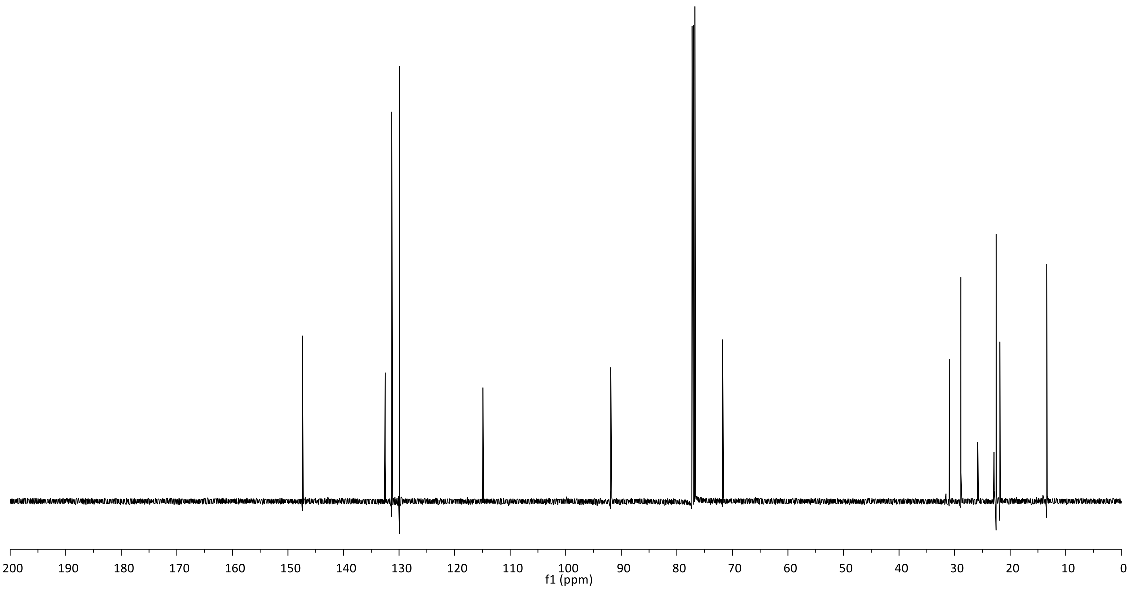
200
190
180
170
160
150
140
130
120
110
100
90
80
70
60
50
40
30
20
10
0
f1 (ppm)

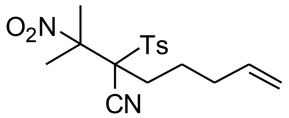
O2N
Ts
CN

3h

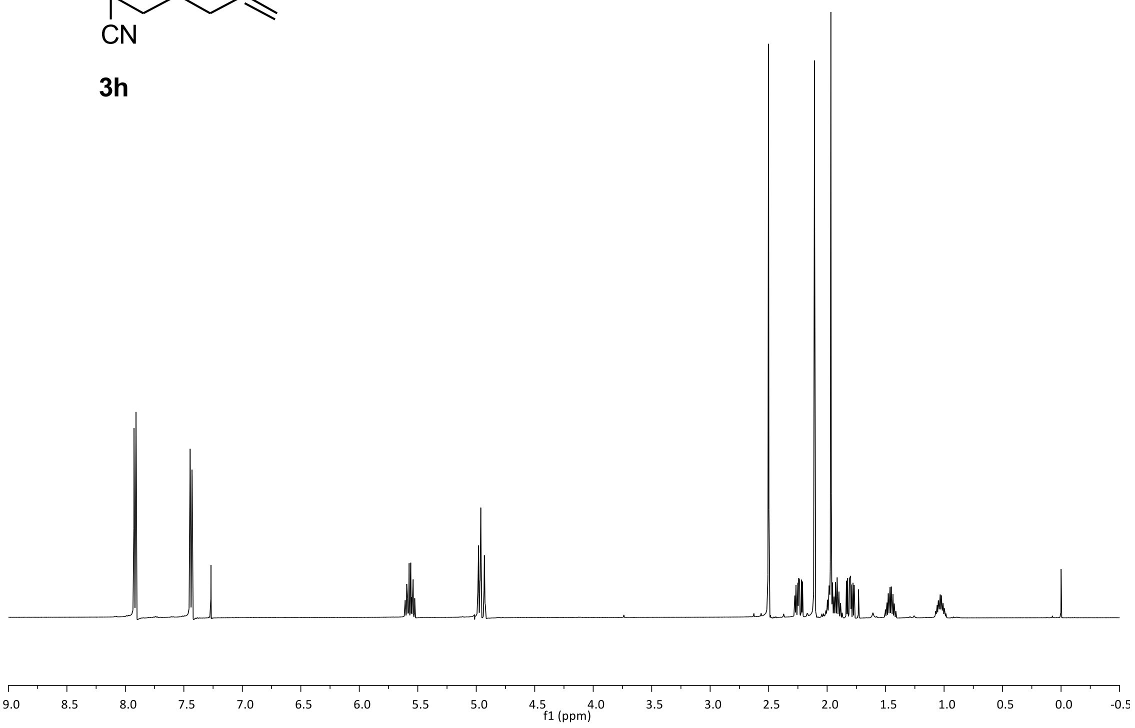
9.0 8.5 8.0 7.5 7.0 6.5 6.0 5.5 5.0 4.5 4.0 3.5 3.0 2.5 2.0 1.5 1.0 0.5 0.0
f1 (ppm)

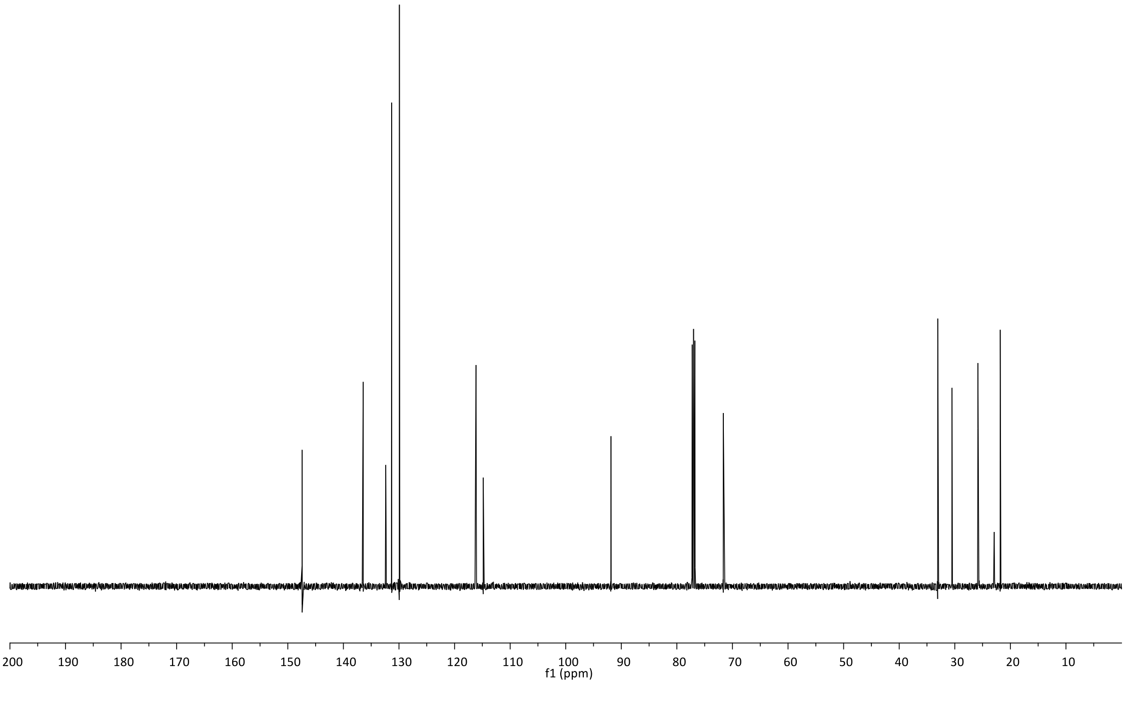
200 190 180 170 160 150 140 130 120 110 100 90 80 70 60 50 40 30 20 10
f1 (ppm)
